# Supplementary material for: Safe spaces for youth mental health: A scoping review
Source: PLoS One. 2025 Apr 4;20(4):e0321074. doi: 10.1371/journal.pone.0321074 (PMC11970664; doi:10.1371/journal.pone.0321074)
Supplement: Supplementary File 1 — (DOCX) [file pone.0321074.s001.docx]

| Ovid MEDLINE(R) ALL <1946 to December 05, 2023> | |  |
| --- | --- | --- |
| 1 | safe space*.mp. | 897 |
| 2 | ("Community center*" or "community centre*" or "leisure center*" or "leisure centre*" or "recreation center*" or "recreation centre*" or "civic center*" or "civic centre*").mp. | 2622 |
| 3 | ("Youth club*" or "boys and girls club*" or "girl scout*" or "girl guides" or brownies or embers or "boy scout*").mp. | 456 |
| 4 | (youth* adj2 program*).mp. | 1841 |
| 5 | Libraries/ | 2525 |
| 6 | ("public librar*" or "school* librar*").mp. | 637 |
| 7 | ("school* based" or "school* intervention*" or "school* program*" or "school* setting*" or "school* space*").mp. | 23663 |
| 8 | (classroom* adj2 (workshop* or education* or training or intervention* or setting* or space* or program*)).mp. | 2990 |
| 9 | ((virtual* or digital* or online* or internet* or web*) adj3 (setting* or environment* or group* or space* or communit*)).mp. | 27962 |
| 10 | ("summer camp*" or "day camp*").mp. | 1257 |
| 11 | ("Neighbo?rhood place*" or "neighbo?rhood space*").mp. | 126 |
| 12 | shelter?.mp. | 10727 |
| 13 | ("drop-in center*" or "drop-in centre*").mp. | 456 |
| 14 | or/1-13 | 74939 |
| 15 | Adolescent/ | 2215665 |
| 16 | Minors/ | 2820 |
| 17 | (adolescen* or teen* or youth? or pre-teen* or preteen* or minor?).mp. | 2570942 |
| 18 | ((social* or emotional* or behavio?ral* or cognitive* or moral*) adj2 (competen* or moral*)).mp. | 61409 |
| 19 | exp Self Concept/ | 122152 |
| 20 | (self-efficacy or self-determination or self-worth).mp. | 57007 |
| 21 | ((clear or positive) adj1 identi*).mp. | 5254 |
| 22 | belie* in the future.mp. | 68 |
| 23 | ("positive norm*" or "positive emotion*" or "positive bond*").mp. | 7254 |
| 24 | exp Mental Health/ OR mental health.tw. OR exp Mental Disorders/ OR (mental disorder* or Mental diseas*).tw. OR Anxiety/ or Performance Anxiety/ OR Catastrophizing/ OR (anxiet* or catastrophiz*).tw. OR Depression*.tw. OR suicide/ or attempted suicide/ or suicidal ideation/ OR suicid*.tw. OR psychological distress*.tw. OR empower*.tw. OR exp Hope/ OR hope*.tw. OR confiden*.tw. OR self-concept/ OR Body Image/ OR Self-Efficacy/ OR Self-Compassion/ OR (self concept* or body image* or self efficac* or self compassion*).tw. OR Self-Esteem/ OR Well Being/ OR (self esteem* or self-esteem* or Long?Term Happiness or wellbeing* or well-being*).tw. | 11032 |
| 25 | active* participat*.mp. | 8243 |
| 26 | or/26-37 | 475176 |
| 27 | 14 and 21 and 37 | 6118 |
| 28 | limit 37 to (english language and yr="2013 -Current") | 1711 |

| Embase <1974 to 2023 December 05> | |  |
| --- | --- | --- |
| 1 | safe space*.mp. | 1273 |
| 2 | ("Community center*" or "community centre*" or "leisure center*" or "leisure centre*" or "recreation center*" or "recreation centre*" or "civic center*" or "civic centre*").mp. | 3882 |
| 3 | ("Youth club*" or "boys and girls club*" or "girl scout*" or "girl guides" or brownies or embers or "boy scout*").mp. | 555 |
| 4 | (youth* adj2 program*).mp. | 2162 |
| 5 | Libraries/ | 28753 |
| 6 | ("public librar*" or "school* librar*").mp. | 623 |
| 7 | ("school* based" or "school* intervention*" or "school* program*" or "school* setting*" or "school* space*").mp. | 27710 |
| 8 | (classroom* adj2 (workshop* or education* or training or intervention* or setting* or space* or program*)).mp. | 3468 |
| 9 | ((virtual* or digital* or online* or internet* or web*) adj3 (setting* or environment* or group* or space* or communit*)).mp. | 36836 |
| 10 | ("summer camp*" or "day camp*").mp. | 1769 |
| 11 | ("Neighbo?rhood place*" or "neighbo?rhood space*").mp. | 133 |
| 12 | shelter?.mp. | 12468 |
| 13 | ("drop-in center*" or "drop-in centre*").mp. | 576 |
| 14 | or/1-13 | 118342 |
| 15 | Adolescent/ | 1800040 |
| 16 | Minors/ | 906 |
| 17 | (adolescen* or teen* or youth? or pre-teen* or preteen* or minor?).mp. | 2253708 |
| 18 | (self-efficacy or self-determination or self-worth).mp. | 1506735 |
| 19 | ((clear or positive) adj1 identi*).mp. | 1629 |
| 20 | belie* in the future.mp. | 16874 |
| 21 | ("positive norm*" or "positive emotion*" or "positive bond*").mp. | 8299 |
| 22 | personal satisfaction/ | 8930 |
| 23 | life satisfaction.mp. | 13090 |
| 24 | active* participat*.mp. | 50420 |
| 25 | or/26-37 | 1983 |
| 26 | 14 and 21 and37 | 46596 |
| 27 | limit 37 to (english language and yr="2013 -Current") | 892 |

| APA PsycInfo <1806 to Decembe Week 4 2023> | |  |
| --- | --- | --- |
|  |  |  |
| 1 | safe space*.mp. | 1255 |
| 2 | ("Community center*" or "community centre*" or "leisure center*" or "leisure centre*" or "recreation center*" or "recreation centre*" or "civic center*" or "civic centre*").mp. | 1799 |
| 3 | ("Youth club*" or "boys and girls club*" or "girl scout*" or "girl guides" or brownies or embers or "boy scout*").mp. | 569 |
| 4 | (youth* adj2 program*).mp. | 3355 |
| 5 | Libraries/ | 976 |
| 6 | ("public librar*" or "school* librar*").mp. | 899 |
| 7 | ("school* based" or "school* intervention*" or "school* program*" or "school* setting*" or "school* space*").mp. | 50953 |
| 8 | (classroom* adj2 (workshop* or education* or training or intervention* or setting* or space* or program*)).mp. | 11135 |
| 9 | ((virtual* or digital* or online* or internet* or web*) adj3 (setting* or environment* or group* or space* or communit*)).mp. | 27237 |
| 10 | ("summer camp*" or "day camp*").mp. | 1312 |
| 11 | ("Neighbo?rhood place*" or "neighbo?rhood space*").mp. | 71 |
| 12 | shelter?.mp. | 6933 |
| 13 | ("drop-in center*" or "drop-in centre*").mp. | 416 |
| 14 | or/1-13 | 103147 |
| 15 | Adolescent/ | 67674 |
| 16 | Minors/ | 606491 |
| 17 | (adolescen* or teen* or youth? or pre-teen* or preteen* or minor?).mp. | 606491 |
| 18 | (self-efficacy or self-determination or self-worth).mp. | 95294 |
| 19 | ((clear or positive) adj1 identi*).mp. | 5901 |
| 20 | belie* in the future.mp. | 9861 |
| 21 | ("positive norm*" or "positive emotion*" or "positive bond*").mp. | 61455 |
| 22 | exp Mental Health/ OR mental health.tw. OR exp Mental Disorders/ OR (mental disorder* or Mental diseas*).tw. OR Anxiety/ or Performance Anxiety/ OR Catastrophizing/ OR (anxiet* or catastrophiz*).tw. OR Depression*.tw. OR suicide/ or attempted suicide/ or suicidal ideation/ OR suicid*.tw. OR psychological distress*.tw. OR empower*.tw. OR exp Hope/ OR hope*.tw. OR confiden*.tw. OR self-concept/ OR Body Image/ OR Self-Efficacy/ OR Self-Compassion/ OR (self concept* or body image* or self efficac* or self compassion*).tw. OR Self-Esteem/ OR Well Being/ OR (self esteem* or self-esteem* or Long?Term Happiness or wellbeing* or well-being*).tw. | 1455 |
| 23 | personal satisfaction/ | 18153 |
| 24 | life satisfaction.mp. | 35385 |
| 25 | active* participat*.mp. | 4950 |
| 26 | or/26-39 | 544 |
| 27 | 14 and 21 and 68 | 4245 |
| 28 | limit 69 to (english language and yr="2013 -Current") | 2726 |

| Database: CINAHL Plus with Full Text via EBSCOhost (1936 - Present) | |  |
| --- | --- | --- |
| Date of search: Decembe 7, 2023 | |  |
|  |  |  |
| S1 | "safe space*" | 1255 |
| S2 | ("Community center*" or "community centre*" or "leisure center*" or "leisure centre*" or "recreation center*" or "recreation centre*" or "civic center*" or "civic centre*") | 1799 |
| S3 | ("Youth club*" or "boys and girls club*" or "girl scout*" or "girl guides" or brownies or embers or "boy scout*") | 569 |
| S4 | (youth* N2 program*) | 3355 |
| S5 | (MH "Libraries") OR (MH "Libraries, Public") OR (MH "Libraries, School") | 976 |
| S6 | ("public librar*" or "school* librar*") | 899 |
| S7 | ("school* based" or "school* intervention*" or "school* program*" or "school* setting*" or "school* space*") | 50953 |
| S8 | (classroom* N2 (workshop* or education* or training or intervention* or setting* or space* or program*)) | 11135 |
| S9 | ((virtual* or digital* or online* or internet* or web*) N3 (setting* or environment* or group* or space* or communit*)) | 27237 |
| S10 | ("summer camp*" or "day camp*") | 1312 |
| S11 | ("Neighbo#rhood place*" or "neighbo#rhood space*") | 71 |
| S12 | shelter# | 6933 |
| S13 | ("drop-in center*" or "drop-in centre*") | 416 |
| S14 | S1 OR S2 OR S3 OR S4 OR S5 OR S6 OR S7 OR S8 OR S9 OR S10 OR S11 OR S12 OR S13 | 103147 |
| S15 | (MH "Adolescence+") OR (MH "Minors (Legal)") | 67674 |
| S16 | (adolescen* or teen* or youth# or pre-teen* or preteen* or minor#) | 606491 |
| S17 | (MH "Adolescent Health") | 606491 |
| S18 | exp Mental Health/ OR mental health.tw. OR exp Mental Disorders/ OR (mental disorder* or Mental diseas*).tw. OR Anxiety/ or Performance Anxiety/ OR Catastrophizing/ OR (anxiet* or catastrophiz*).tw. OR Depression*.tw. OR suicide/ or attempted suicide/ or suicidal ideation/ OR suicid*.tw. OR psychological distress*.tw. OR empower*.tw. OR exp Hope/ OR hope*.tw. OR confiden*.tw. OR self-concept/ OR Body Image/ OR Self-Efficacy/ OR Self-Compassion/ OR (self concept* or body image* or self efficac* or self compassion*).tw. OR Self-Esteem/ OR Well Being/ OR (self esteem* or self-esteem* or Long?Term Happiness or wellbeing* or well-being*).tw. |  |
| S19 | S14 OR S15 OR S16 OR S17 OR S18 OR S19 OR S20 OR S21 OR S22 OR S23 | 544 |
| S20 | S14 OR S18 | 1799 |
| S21 | (S14 AND S224 AND S24): Limit to pub date 2013-2023, English language, peer-reviewed journals | 1960 |

Scopus

| Database: Scopus (1976 - Present) |  |
| --- | --- |
| Date of search: Decembe, 2023 |  |
|  |  |
| TITLE-ABS-KEY ( ( ( child* OR underage* OR minor* OR adolescen* OR teen ) W/2 ( marriage OR marry OR married OR marries ) ) ) ) ) ) AND ( TITLE-ABS-KEY ( ( adolescen* OR teen* OR youth? OR pre-teen* OR preteen* OR minor? ) ) ) AND ( ( ( TITLE-ABS-KEY ( ( "Neighbo?rhood place*" OR "neighbo?rhood space*" ) ) OR TITLE-ABS-KEY ( shelter? OR "drop-in center*" OR "drop-in centre*" ) ) ) OR ( ( TITLE-ABS-KEY ( "safe space*" ) OR TITLE-ABS-KEY ( ( "Community center*" OR "community centre*" OR "leisure center*" OR "leisure centre*" OR "recreation center*" OR "recreation centre*" OR "civic center*" OR "civic centre*" ) ) OR TITLE-ABS-KEY ( ( "Youth club*" OR "boys and girls club*" OR "girl scout*" OR "girl guides" OR brownies OR embers OR "boy scout*" ) ) OR TITLE-ABS-KEY ( ( youth* W/2 program* ) ) OR TITLE-ABS-KEY ( ( "public librar*" OR "school* librar*" ) ) OR TITLE-ABS-KEY ( ( "school* based" OR "school* intervention*" OR "school* program*" OR "school* setting*" OR "school* space*" ) ) OR TITLE-ABS-KEY ( ( classroom* W/2 ( workshop* OR education* OR training OR intervention* OR setting* OR space* OR program* ) ) ) exp Mental Health/ OR mental health.tw. OR exp Mental Disorders/ OR (mental disorder* or Mental diseas*).tw. OR Anxiety/ or Performance Anxiety/ OR Catastrophizing/ OR (anxiet* or catastrophiz*).tw. OR Depression*.tw. OR suicide/ or attempted suicide/ or suicidal ideation/ OR suicid*.tw. OR psychological distress*.tw. OR empower*.tw. OR exp Hope/ OR hope*.tw. OR confiden*.tw. OR self-concept/ OR Body Image/ OR Self-Efficacy/ OR Self-Compassion/ OR (self concept* or body image* or self efficac* or self compassion*).tw. OR Self-Esteem/ OR Well Being/ OR (self esteem* or self-esteem* or Long?Term Happiness or wellbeing* or well-being*).tw. OR TITLE-ABS-KEY ( ( ( virtual* OR digital* OR online* OR internet* OR web* ) W/3 ( setting* OR environment* OR group* OR space* OR communit* ) ) ) OR TITLE-ABS-KEY ( ( "summer camp*" OR "day camp*" ) ) ) ) ) AND PUBYEAR > 2012 AND PUBYEAR < 2024 AND ( LIMIT-TO ( DOCTYPE , "ar" ) OR LIMIT-TO ( DOCTYPE , "re" ) ) AND ( LIMIT-TO ( LANGUAGE , "English" ) ) | 1160 |
