# Supplementary material for: Safe spaces for youth mental health: A scoping review
Source: PLoS One. 2025 Apr 4;20(4):e0321074. doi: 10.1371/journal.pone.0321074 (PMC11970664; doi:10.1371/journal.pone.0321074)
Supplement: Attachment — Submitted filename: Rebuttal Letter-Safe space mental health.docx [file pone.0321074.s004.docx]

**Rebuttal letter**

Dear Dr. Nisa,

Thank you for submitting your manuscript to PLOS ONE. After careful consideration, we feel that it has merit but does not fully meet PLOS ONE’s publication criteria as it currently stands. Therefore, we invite you to submit a revised version of the manuscript that addresses the points raised during the review process.

The authors have presented a systematic review and the writing does bring forth the key concepts and the characteristics of the studies in the given domain of safe spaces and the interventions used in such spaces with some evidence of effect on mental health outcomes. While a detailed analysis is out of scope for this review, even for a scoping review the authors should attempt to classify in the introduction as well as in the abstract as what they classified as safe spaces. This is more clear from looking at the search terms in the supplement but it would benefit the reader to know from abstract itself as to what is the main domain of this review.

The reviewers have pointed out some language errors which the authors should address. The authors have highlighted some knowledge gaps specific to how the research is less in some countries in this domain and how longitudinal research may add further. Yet these are more general limitations present in most of the literature considering a larger number of studies are cross-sectional as well as this domain is not so well researched. It would serve better to expand on how the studies could have improved and what are the barriers that could help in more research in this area, such as whether it is more of an awareness issue related to safe space, or there is less attention given to mental health. Expanding on the knowledge gaps would make the review more meaningful.

Please submit your revised manuscript by Jan 31 2025 11:59PM. If you will need more time than this to complete your revisions, please reply to this message or contact the journal office at plosone@plos.org. When you're ready to submit your revision, log on to <https://www.editorialmanager.com/pone/> and select the 'Submissions Needing Revision' folder to locate your manuscript file.

Response: Thank you for your detailed feedback and the opportunity to revise our manuscript. We addressed all the points raised, including clarifying the classification of safe spaces in the abstract and introduction, correcting language errors, and expanding on knowledge gaps and barriers in this research area.Please see the color change in the revised paper

Please include the following items when submitting your revised manuscript:

- A rebuttal letter that responds to each point raised by the academic editor and reviewer(s). You should upload this letter as a separate file labeled 'Response to Reviewers'.
- A marked-up copy of your manuscript that highlights changes made to the original version. You should upload this as a separate file labeled 'Revised Manuscript with Track Changes'.
- An unmarked version of your revised paper without tracked changes. You should upload this as a separate file labeled 'Manuscript'.

Response: Dear editor, Thank you for your feedback and the opportunity to revise our manuscript. We addressed all points raised, revising the abstract and introduction to clearly define "safe spaces," correcting language errors, and expanding the discussion on knowledge gaps by detailing study improvements and barriers to research. We have uploaded a rebuttal letter, a revised manuscript with tracked changes, and a clean version without tracked changes. Thank you for your guidance, and we look forward to your response.

If you would like to make changes to your financial disclosure, please include your updated statement in your cover letter. Guidelines for resubmitting your figure files are available below the reviewer comments at the end of this letter.

If applicable, we recommend that you deposit your laboratory protocols in protocols.io to enhance the reproducibility of your results. Protocols.io assigns your protocol its own identifier (DOI) so that it can be cited independently in the future. For instructions see: <https://journals.plos.org/plosone/s/submission-guidelines#loc-laboratory-protocols>. Additionally, PLOS ONE offers an option for publishing peer-reviewed Lab Protocol articles, which describe protocols hosted on protocols.io. Read more information on sharing protocols at <https://plos.org/protocols?utm_medium=editorial-email&utm_source=authorletters&utm_campaign=protocols>.

We look forward to receiving your revised manuscript.

Kind regards,

Aditya Pawar
Guest Editor
PLOS ONE

Journal requirements:

When submitting your revision, we need you to address these additional requirements.

1. Please ensure that your manuscript meets PLOS ONE's style requirements, including those for file naming. The PLOS ONE style templates can be found at

https://journals.plos.org/plosone/s/file?id=wjVg/PLOSOne_formatting_sample_main_body.pdf and

<https://journals.plos.org/plosone/s/file?id=ba62/PLOSOne_formatting_sample_title_authors_affiliations.pdf>

Response:Dear Editor, Thank you so mucch. We addressed the additional requirements by ensuring the manuscript complied with PLOS ONE's style guidelines

2. As required by our policy on Data Availability, please ensure your manuscript or supplementary information includes the following:

A numbered table of all studies identified in the literature search, including those that were excluded from the analyses.

For every excluded study, the table should list the reason(s) for exclusion.

If any of the included studies are unpublished, include a link (URL) to the primary source or detailed information about how the content can be accessed.

A table of all data extracted from the primary research sources for the systematic review and/or meta-analysis. The table must include the following information for each study:

Name of data extractors and date of data extraction

Confirmation that the study was eligible to be included in the review.

All data extracted from each study for the reported systematic review and/or meta-analysis that would be needed to replicate your analyses.

Response :We have fulfilled the Data Availability requirements by including a table of all studies from the literature search, with reasons for exclusion where applicable. For unpublished studies, we provided URLs or access details. Additionally, we included a data extraction table listing extractor names, extraction dates, study eligibility confirmation, and all data required to replicate the analyses.

If applicable for your analysis, a table showing the completed risk of bias and quality/certainty assessments for each study or outcome. Please ensure this is provided for each domain or parameter assessed. For example, if you used the Cochrane risk-of-bias tool for randomized trials, provide answers to each of the signalling questions for each study. If you used GRADE to assess certainty of evidence, provide judgements about each of the quality of evidence factor. This should be provided for each outcome.

An explanation of how missing data were handled.

This information can be included in the main text, supplementary information, or relevant data repository. Please note that providing these underlying data is a requirement for publication in this journal, and if these data are not provided your manuscript might be rejected.

Response: Thank you so much for your insights. However, We did not assess the quality of the studies in the scoping reviews is not mandatory. Moreover, our focus was on mapping the existing evidence rather than assessing the quality of the studies, ensuring alignment with the objectives of a scoping study.

Reviewers' comments:

Reviewer's Responses to Questions

**Comments to the Author**

1. Is the manuscript technically sound, and do the data support the conclusions?

The manuscript must describe a technically sound piece of scientific research with data that supports the conclusions. Experiments must have been conducted rigorously, with appropriate controls, replication, and sample sizes. The conclusions must be drawn appropriately based on the data presented.

Reviewer #1: Yes

Reviewer #2: Partly

Reviewer #3: Yes

Reviewer #4: Yes

Response: Dear all reviewers, We appreciate your feedback. While Reviewers #1, #3, and #4 confirmed the manuscript's technical soundness and data support, we hope we have addressed Reviewer #2's concerns.

2. Has the statistical analysis been performed appropriately and rigorously?

Reviewer #1: I Don't Know

Reviewer #2: N/A

Reviewer #3: Yes

Reviewer #4: I Don't Know

Response: Dear all reviewers ,Thank you for your feedback.

3. Have the authors made all data underlying the findings in their manuscript fully available?

The [PLOS Data policy](http://www.plosone.org/static/policies.action#sharing) requires authors to make all data underlying the findings described in their manuscript fully available without restriction, with rare exception (please refer to the Data Availability Statement in the manuscript PDF file). The data should be provided as part of the manuscript or its supporting information, or deposited to a public repository. For example, in addition to summary statistics, the data points behind means, medians and variance measures should be available. If there are restrictions on publicly sharing data—e.g. participant privacy or use of data from a third party—those must be specified.

Reviewer #1: Yes

Reviewer #2: Yes

Reviewer #3: Yes

Reviewer #4: Yes

Response: We appreciate the reviewers' confirmation that all underlying data are fully available

4. Is the manuscript presented in an intelligible fashion and written in standard English?

PLOS ONE does not copyedit accepted manuscripts, so the language in submitted articles must be clear, correct, and unambiguous. Any typographical or grammatical errors should be corrected at revision, so please note any specific errors here.

Reviewer #1: Yes

Reviewer #2: Yes

Reviewer #3: Yes

Reviewer #4: Yes

Response: We are pleased that all reviewers confirmed the manuscript is presented clearly and written in standard English. We have ensured that the language is clear, correct, and unambiguous, with no typographical or grammatical errors.

5. Review Comments to the Author

Reviewer #1: The manuscript is well written in a lucid style and highlights an important aspect of emotional ,social and holistic development of youths and young adults. More research and actions need to be undertaken in the community development of safe spaces.

Response: Dear reviewer, we appreciate your positive feedback on our manuscript and agree that further research and action are needed to develop safe spaces for youths and young adults in community settings.

Reviewer #2: Thank you for the opportunity to review this paper. This manuscript tackles an important topic by exploring the role of safe spaces in supporting youth mental health. It addresses a critical area of research with global significance. Here are my comments and suggestions:

Response: We thank you for your positive feedback and recognition of the importance of our research. We appreciate your valuable comments and suggestions, which we carefully addressed in colour change in the main document and as described below.

Abstract: Under method section - “Followed”, make it lower case to “followed”

Response: Thank you for this feedback. As per your suggestion, the word "Followed" in the method section has been changed to lowercase. Kindly see the colour change.

- Introduction: Make it consistent “mental health” where possible. I see to many different terminology used for the same.
- “Mental health disorders in youth include a range of conditions such as depression, anxiety disorders, eating disorders, substance use disorders, attention-deficit/hyperactivity disorder (ADHD), and post traumatic stress disorder (PTSD)”. Consider adding schizophrenia, as first episode psychosis and diagnosis of Schizophrenia tends to be very common in age group between 15-25 years old.

Response: Dear reviewer, Thanks for this critical input. The terminology in the introduction has been made consistent by using “mental health” throughout, where applicable. We have also modified the suggested inputs. Kindly see the colour change.
- Search Strategy : Authors indicated that search for Grey literatures was done. Explain if those literatures were used for the review or excluded. If used then I recommend to indicate separately in the PRISMA flow chart.

Response: Dear Reviewer, Thank you for your feedback. As indicated in the reference section, we have searched the grey literature as suggested and have already described it separately. Three grey literature sources were included. Please refer to the PRISMA flow diagram for further details.

- Data Extraction : Please include details on how you handled missing data in the included studies during extraction.

Response: thank you for your feedback. We have included details on how we handled missing data in this scoping review. Please see the highlighted changes in the main document.

- Results and discussion :You mentioned the imbalance in geographical distribution earlier in the paper. Consider providing a comparative analysis of interventions across regions to offer deeper insights into the influence of regional differences on intervention efficacy.

Response: Thank you for the insightful feedback regarding the geographical imbalance. As this is a scoping review, its primary aim is to map existing evidence and identify gaps rather than perform detailed comparative analyses. While we have noted the regional differences and their implications in our findings, conducting an in-depth analysis of intervention efficacy across regions is beyond the scope of this review. However, such an analysis would offer valuable insights and recommend future systematic reviews or meta-analyses to explore these regional variations in depth.Thank you for your feedbacks

- Consider adding recommendations for funding frameworks and longitudinal study designs to ensure the sustainability and long-term impact of these interventions.

Response: We have added recommendations for funding frameworks and longitudinal study designs in the discussion to ensure these interventions' sustainability and long-term impact at the implication section.
- The manuscript effectively highlights schools as key environments for youth mental health interventions but only briefly mentions community-based interventions. Consider addressing how community settings could fill gaps left by school-based programs, such as supporting out-of-school youth.

Response: We expanded the discussion to highlight how community settings can fill gaps left by school-based interventions, particularly in supporting out-of-school youth.

Reviewer #3: Overall, this is a well-written article. It provides a good case for the need to explore safe spaces and its overall benefit to youth.

Response: We appreciate the positive feedback and are glad the article effectively conveys the importance of exploring safe spaces and their benefits to youth.

Introduction: This section includes a good review of research already present on the topic, along with defining safe spaces and the myriad of environments they can be present. Some considerations can be made with the wording. For example, for the following sentence on page 3: “Mental ill-health is a significant challenge, posing a threat to individuals’ health, well-being, …” It appears redundant to mention mental ill-health and then an individuals’ health again in the same sentence. Also, what is the reasoning behind mentioning “mental ill-health” vs. calling it “mental illness”?

Further along in the same paragraph, it mentions: “Youth mental health programs such as mentoring initiatives, collaborative mental health promotion, and friends for life initiatives…” It seems that the “friends for life” initiative should be capitalized as it seems to be naming a specific initiative.

Response: Thank you for this feedback. We have revised the document to address your concerns. Please see the highlighted changes.

**Materials and Methods:
Authors have done a good job of providing a thorough explanation of the methods used in the review. They can consider a table to synthesize and categorize the final set of studies chosen for the review. For example, how many were initially reviewed, how many were selected and of those how many were school-based, community-based, etc. Providing it in a table or figure format would make visualization easier for the reader.**

**Response: Thank you so much for this critical feedback. We have used a PRISMA diagram to outline the study selection process, and the suggestion for tables has been incorporated into the Results section following the Joanna Briggs Institute (JBI) scoping review guidelines.Kindly see the changes made in the main document.**

Under eligibility criteria on page 4, it mentions “The review examines mental health outcomes like decreased depression, anxiety, stress, resilience, and self-esteem among youth in safe spaces.” Authors can consider omitting the word “decreased” or adding the word “increased” before “resilience, and self-esteem” for consistency.
Response: We have revised the sentence under eligibility criteria for consistency per your golden feedback. See the colour change in the main document.
On page 6, under “features of the included studies” section, it states: “North America emerges as the most represented region…followed by Europe…including countries like Norway…”. The word “like” is not necessary, authors can simply list the countries that were included without the word “like”.

Response: Thank you for your feedback. We have revised the sentence based on your feedback. See the colour change in the main document.

On page 7, where “FRIENDS for Life initiative” is mentioned, it would be helpful to provide brief information about what that entails.

Response: Thank you for the suggestion. We have updated page 7 to briefly explain the "FRIENDS for Life" initiative, highlighting its focus on building resilience and promoting social and emotional skills to support mental health. See also the color change.

Mental Health Outcomes:
It is to note that the body of the review does not have a clearly labelled “Results” section. Authors can consider adding this.

On page 8, it mentions “Mental health and well-being outcomes include reductions in PTSD, anxiety, gambling symptoms, and a decrease in intrusive thoughts”. Please clarify what is meant by intrusive thoughts – was the study looking at OCD, or intrusive thoughts of a suicidal nature, etc.?

Response:We have added a clearly labelled Results section to the body of the review as suggested. Regarding the mention of “intrusive thoughts,” we have clarified that it refers to those related to OCD or suicidal ideation, depending on the study.

Although it is a scoping review, authors can consider mentioning some specific outcomes, such as the range of decrease in depressive symptoms across various studies (e.g. 10-50%), or the range of decrease in anxiety or suicidal thoughts, etc. It would also be interesting to note any differences in outcomes between safe spaces in schools vs. community centers vs. primary care centers, etc.
Response: We appreciate your valuable suggestion. As this is a scoping review, our aim was to map the existing evidence rather than synthesize it. However, synthesizing the findings could provide more detailed insights. Therefore, we recommend that future research focus on synthesizing these outcomes, either through a systematic review or meta-analysis, as suggested.
Discussion:
This section provided a good review of the results, its applicability to the public, and future considerations for research. On page 10, it mentions: “Nevertheless, with valuable insights into mental health intervention within safe spaces, our stay may have limitations”. This appears to be a typo, I believe it meant to say “..our study has some limitations”.
Response:Thank you for pointing this out. We have corrected the typo, and the revised sentence now reads: “Nevertheless, with valuable insights into mental health intervention within safe spaces, our study has some limitations.”
Overall, the paper did a good job at highlighting areas of growth in the community for improving mental health in youth. Authors can consider the points discussed above.

Response: Thank you for your feedback. We appreciate your recognition of the paper's strengths and have carefully considered the above points to improve the manuscript.Thank you so much for your critical inputs.

Reviewer #4: The scoping review titled "Safe Spaces for Youth Mental Health" provides a valuable synthesis of the literature concerning safe spaces and their role in enhancing youth mental health. While the limitations have been discussed, there are some additional limitations that need to be addressed or mentioned in the limitations:
1. The majority of included studies originate from high-income regions (e.g., North America and Europe), with limited representation from low- and middle-income countries.
2. Although the study targets ages 10-25, the majority of interventions cater to younger adolescents, neglecting older youth.
3. Intersectional factors such as gender, ethnicity, and socioeconomic status are underexplored.
4. While the study emphasizes implications for policy and practice, specific actionable recommendations are limited.

Response: Thank you so much for this critical feedback. As you described, the above-discussed issues are gaps in this scoping review. We have addressed these gaps in the implications section and provided future directions based on the identified gaps. Kindly see the revised version with the colour change. Thank you once again for your valuable insights!

We sincerely thank all the reviewers and editors for their valuable feedback and insightful suggestions. Your thoughtful comments have significantly enhanced the quality and clarity of our manuscript. We deeply appreciate your time and effort in reviewing our work and providing constructive recommendations. Thank you for guiding us in refining our research and addressing critical areas to strengthen the paper.

Saba

On behalf coauthors
