# Supplementary material for: Safe spaces for youth mental health: A scoping review
Source: PLoS One. 2025 Apr 4;20(4):e0321074. doi: 10.1371/journal.pone.0321074 (PMC11970664; doi:10.1371/journal.pone.0321074)
Supplement: Attachment — Submitted filename: Rebuttal Letter 27-02-2025.docx [file pone.0321074.s005.docx]

**Dear Reviewers and Editors,**

We have made the required changes and carefully addressed the points you raised in your feedback. We appreciate your time and thoughtful suggestions, which have helped us improve the manuscript.

**Reviewer #3:** Authors have done a good job with the revision of the paper. Authors can consider the following edits:
1. On page 3, it states “Mental health is a significant challenge, posing a threat to individuals’ health, well-being…”. Authors can consider changing this to “Mental illness is a significant challenge to an individual’s overall health, well-being, and productivity.” Reasoning would be that it is mental illness, not mental health, that is the challenge. Also, mentioning the word “health” twice in the same sentence appears redundant.

Thank you so much for highlighting the necessary changes. We have changed it to the suggested sentence.

2. On page 7, please clarify if “FRIENDS for Life” should be capitalized as such? In the introduction section earlier, it is capitalized as “Friends for Life”. Please make it is consistent throughout the article.

Thank you for highlighting this error, we have made it consistent.
